# High‐Altitude Hypoxia Activates JNK‐p53 Signaling: Linking Hippocampal Energy Crisis to Cognitive Impairment

**DOI:** 10.1002/cns.70986

**Published:** 2026-06-17

**Authors:** Guisheng Hao, Zhengzhong Bai, Wenjuan Wang, Jian Wu, Fan Zheng, Guoen Jin, Ri‐Li Ge

**Affiliations:** ^1^ Research Center for High Altitude Medicine Qinghai University, Key Laboratory of the Ministry of High Altitude Medicine, Laboratory for High Altitude Medicine of Qinghai Province, Key Laboratory of Applied Basic Research in High Altitude Medicine Xining China; ^2^ Qinghai Provincial People's Hospital Xining China; ^3^ Beijing Tsinghua Changgung Hospital, School of Clinical Medicine Tsinghua University Beijing China

**Keywords:** cognitive impairment, energy metabolism, high‐altitude hypoxia, JNK‐p53 axis, mitochondrial dysfunction

## Abstract

**Background:**

Chronic high‐altitude hypoxia impairs hippocampal memory, yet population‐level dose–response relationships and the molecular mechanisms linking hypoxic stress to energy metabolic collapse remain poorly defined.

**Methods:**

We conducted a cross‐sectional study of 2819 residents (living at altitudes between 3000 and 5000 m). In parallel, we established a rat model of sustained hypobaric hypoxia (6000 m, 1–28 days) and applied pharmacological intervention using the JNK inhibitor JNK‐IN‐8. Metabolomic profiling, transmission electron microscopy, and molecular analyses were performed to assess metabolic reprogramming, mitochondrial ultrastructure, and signaling pathways.

**Results:**

Residents at > 4000 m exhibited 91% higher memory impairment risk. Chronic hypoxia activated JNK‐p53‐Bim signaling, driving mitophagy‐to‐apoptosis transition, mitochondrial cristae disruption, and 73% ATP depletion by Day 28. JNK‐IN‐8 partially restored ATP and suppressed p53. HIF‐2α/PHD2 colocalization indicated parallel adaptive signaling.

**Conclusions:**

Chronic hypoxia induces memory impairment via JNK‐mediated mitochondrial dysfunction. JNK inhibition offers therapeutic potential, while concurrent HIF‐2α/PHD2 activation suggests a complex balance between hypoxic injury and adaptation.

## Graphical abstract

Chronic high‐altitude hypoxia impairs memory in 2819 residents and causes hippocampal atrophy in rats. JNK‐p53‐Bim signaling drives mitophagy‐to‐apoptosis transition and 73% ATP depletion, while concurrent HIF‐2α/PHD2 activation indicates parallel adaptive responses. JNK inhibition partially restores ATP, establishing this axis as a therapeutic target for hypoxia‐induced cognitive decline.

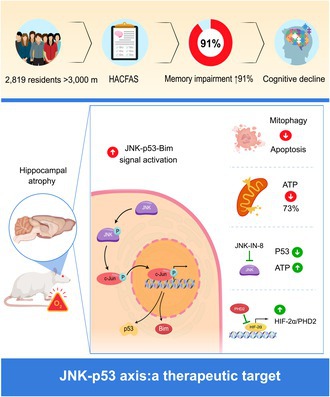

## 1 Introduction

Globally, more than 500 million individuals reside chronically at altitudes of at least 3000 m [1], where hypobaric hypoxia poses a particular threat to the brain due to its high metabolic demand and exquisite sensitivity to oxygen deprivation. Prolonged exposure can lead to irreversible structural damage in the hypoxia‐vulnerable hippocampus, resulting in persistent learning and memory dysfunction [2, 3]. The cognitive consequences of such dysfunction impose substantial but under‐recognized burdens on both individuals and society [4, 5]. Memory dysfunction not only compromises daily functioning and quality of life for permanent highland residents but also elevates the long‐term risk for neurodegenerative diseases such as Alzheimer's disease [6]. At the societal level, these deficits threaten occupational safety in high‐altitude industries (e.g., mining, military operations) [7] and impose significant public healthcare costs. However, population‐level dose–response relationships between altitude exposure and specific cognitive impairments remain poorly defined.

High‐altitude hypobaric hypoxia disrupts neuronal energy metabolism [8]. Although hypoxia‐inducible factor‐1α (HIF‐1α) induces glycolytic compensation [9], hippocampal ATP levels decline following 24 h of sustained hypoxia [10], suggesting the involvement of HIF‐independent regulatory pathways. However, the upstream signaling pathways that trigger mitochondrial dysfunction and energy metabolic collapse remain elusive.

The c‐Jun N‐terminal kinase (JNK) is a stress‐activated protein kinase responsive to hypoxia and oxidative stress, serving as a critical node that detects hypoxic injury and amplifies associated damage signals [11]. Upon activation, JNK phosphorylates p53 and upregulates pro‐apoptotic protein Bim, thereby inducing mitochondrial apoptosis [12]. In addition, JNK mediates tau hyperphosphorylation, which directly downregulates the expression and activity of mitochondrial complexes I and V, consequently suppressing ATP production [13]. JNK inhibition improves behavioral outcomes and attenuates ATP depletion in hypoxia‐ischemia models [14, 15]. These findings collectively indicate a central role for JNK signaling in energy metabolic imbalance. However, whether JNK functions as the molecular switch connecting chronic hypobaric hypoxia to hippocampal energy metabolic crisis and cognitive impairment remains to be determined.

Currently, no pharmacological interventions are approved for chronic high‐altitude cognitive impairment, highlighting the need to validate druggable targets such as the JNK pathway. We hypothesized that JNK‐p53 signaling represents a therapeutic axis linking chronic hypoxic stress to hippocampal energy failure and memory impairment. We aimed to assess population‐level risk across altitude ranges and investigate whether JNK mediates hippocampal mitochondrial dysfunction and energy collapse. Using population epidemiology, longitudinal animal modeling, and JNK inhibitor validation, we demonstrate HIF‐2α/pan‐JNK coordination, progressive mitochondrial dysfunction, and partial therapeutic rescue. While isoform‐specific roles remain unresolved, these findings establish proof‐of‐concept for JNK‐targeted interventions and potential metabolic biomarkers.

## 2 Method and Materials

### 2.1 Study Population and Baseline Assessment

Between 2023 and 2025, we conducted a cross‐sectional survey of long‐term residents on the Qinghai–Tibet Plateau living at altitudes exceeding 3000 m. This study employed an internal reference design wherein the 3000–4000 m group served as a lower‐exposure reference to evaluate the incremental cognitive risk associated with extreme altitude (> 4000 m). Eligibility criteria were as follows: (i) age ≥ 40 years; (ii) continuous residence for ≥ 10 years; and (iii) ability to complete cognitive assessments. Exclusion criteria were as follows: (i) neuropsychiatric disorders, confirmed by medical records from secondary or tertiary hospitals; (ii) missing data exceeding 10% of questionnaire items; and (iii) extreme cognitive assessment results (total scores < 15 or 30).

The High‐Altitude Cognitive Function Assessment Scale is a culturally adapted cognitive screening instrument, developed by our research center (National Copyright Registration No. 2024‐A‐00215453) and validated in 713 residents [16], with detailed psychometric data provided in File S1.

Data were collected onsite by trained research assistants and included demographic information, medical history, and cognitive assessments using the HACFAS.

### 2.2 Cognitive Assessment

Cognitive function was assessed using the High‐Altitude Cognitive Function Assessment Scale (HACFAS), a culturally adapted 30‐point screening instrument encompassing seven domains: delayed word recall, orientation, daily living ability, attention and calculation, language, and visuospatial/executive function. For analytical purposes, scores were grouped into a Memory subscale (delayed recall, 0–5 points) and Non‐memory subscales (all remaining domains, 0–25 points). Memory impairment was operationally defined as a Memory subscale score ≤ 3 (i.e., ≤ 3/5 words recalled). This threshold was selected to ensure measurable item loss for deficit pattern analysis, representing impaired performance clearly distinct from both perfect recall (5/5) and the global impairment exclusion criterion (< 15/30). Participants with total scores < 15 were excluded due to severe impairment or poor compliance. Those with total scores of 30 were excluded due to inability to characterize deficit patterns.

### 2.3 Animals

Eighty male Sprague–Dawley rats (6 weeks old) were obtained from Xi'an Huaren Biotechnology Co. Ltd. and housed under standard laboratory conditions. Following acclimation, rats were randomly assigned to 10 parallel groups (*n* = 8): normoxia control and hypoxia exposure at 1, 7, 14, 21, and 28 days. Only male rats were used to minimize hormonal variability.

### 2.4 Chronic Hypoxia Exposure Protocol

Rats were housed in a hypobaric hypoxic chamber (DYC‐3000) at a simulated altitude of 6000 m (barometric pressure 46.02 kPa, O_2_ partial pressure 8.93 kPa, 24.1°C, 43.5% relative humidity, CO_2_ 2104 ppm) for 28 days and monitored daily. The chamber was briefly decompressed for routine maintenance. On days 1, 7, 14, 21, and 28, rats were removed for Morris water maze testing and euthanized for tissue collection.

### 2.5 Morris Water Maze Experiment

Spatial learning and memory were assessed using the Morris water maze (120 cm diameter, 23°C ± 1°C) with a hidden platform (8 cm diameter, submerged 2 cm below surface). The pool was divided into four quadrants with visual cues. Movements were recorded using EthoVision XT software (Noldus).

The protocol included a 5‐day acquisition phase (four 60‐s trials per day, 20‐min intertrial interval) and probe tests on Days 1, 7, 14, 21, and 28. During probe trials (platform removed), rats were released from the opposite quadrant for 120 s. Platform crossings and target quadrant dwell time were recorded.

### 2.6 Histological Preparation

Rats were anesthetized with urethane (7 mL/kg). Cardiac perfusion was performed with 0.9% saline followed by 4% paraformaldehyde. Brains were removed, post‐fixed in 4% paraformaldehyde for 24 h, dehydrated, and embedded in paraffin. Coronal sections (5 μm) were cut and stained with hematoxylin and eosin (H&E) for examination.

### 2.7 Immunofluorescence Analysis

Paraffin‐embedded coronal brain sections (4 μm) were deparaffinized, rehydrated, and subjected to antigen retrieval. Sections were incubated with primary antibodies (anti‐PHD2 [1:8000, Servicebio, Cat# GB111030], anti‐HIF‐2α [1:5000, Servicebio, Cat# GB111864], anti‐LAMP1 [1:500, Servicebio, Cat# GB112949], and anti‐COX IV [1:500, Servicebio, Cat# GB15250]) at 4°C overnight, followed by HRP‐conjugated secondary antibodies and tyramide signal amplification. Nuclei were counterstained with DAPI and mounted. Images were acquired by fluorescence microscopy and analyzed with QuPath.

### 2.8 Western Blot Analysis of Hippocampal Tissue

Hippocampal tissues were homogenized in RIPA lysis buffer containing protease and phosphatase inhibitors. Protein concentrations were determined using the BCA assay. Samples (20 μg protein per well) were denatured, separated by SDS‐PAGE, and transferred to PVDF membranes. Membranes were blocked and incubated with primary antibodies: HIF‐2α (1:1000, Cat# 57921, Cell Signaling Technology), JNK3 (1:1000, Cat# 2305, Cell Signaling Technology), p‐JNK (1:3000, Cat# AP0631, Abclonal), p‐P53 (1:1000, Cat# 12571, Cell Signaling Technology), BIM (1:1000, Cat# bs 1488R, Bioss Antibodies), OXPHOS cocktail (1:250, Cat# ab110413, Abcam), and β‐actin (1:8000, Cat# 205361AP, Proteintech). After washing, membranes were incubated with HRP‐conjugated secondary antibodies (1:10,000). Protein bands were visualized using enhanced chemiluminescence and quantified using AlphaEaseFC software. Target protein levels were normalized to β‐actin.

### 2.9 Western Blot Analysis of Cell Lysates

Cells were lysed in RIPA buffer, centrifuged (12,000 g, 5 min, 4°C), and supernatants collected. Protein concentrations were measured by BCA assay, and 40 μg protein per well was loaded. Subsequent steps were performed as described for hippocampal tissue lysates.

### 2.10 Metabolomics Analysis

Hippocampal tissue (50 mg) was homogenized in methanol: water (4:1), sonicated, and centrifuged (16,000 g, 20 min, 4°C). Supernatants were dried and reconstituted in methanol: water (1:1). Chromatographic separation was performed using a Shimadzu Nexera X2 UHPLC system with a Waters ACQUITY HSS T3 column. The mobile phase consisted of 0.1% formic acid in water (A) and acetonitrile (B) using a stepwise gradient (0%–48%–100% B).

Mass spectrometric analysis was conducted using a Thermo Q Exactive Plus system in positive and negative ion modes (full scan, m/z 75–1050, 70,000 resolution; data‐dependent MS/MS).

Raw data were processed using MSDIAL. Metabolites were identified against HMDB, MassBank, GNPS, and an in‐house library. Differential metabolites were identified using VIP score > 1.5 and FDR‐adjusted *p* < 0.05.

### 2.11 Transmission Electron Microscopy (TEM)

Hippocampal tissues (1 mm^3^) were fixed with 4% glutaraldehyde and 1% osmium tetroxide, dehydrated in an ethanol–acetone series, embedded in EMBed 812 resin, and polymerized at 60°C for 48 h. Sections (60–80 nm) were cut using a Leica UC7 ultramicrotome, stained with uranyl acetate and lead citrate, and examined using a Hitachi HT7800 transmission electron microscope.

### 2.12 Cell Culture and Hypoxia Treatment

HT22 mouse hippocampal neuronal cells (CL 0697, Wuhan Procell) were maintained in Dulbecco's Modified Eagle Medium at 37°C with 5% CO_2_. Experiments used cells at passages 3–10, seeded at 1 × 10^6^ cells/well in six‐well plates and cultured to 70%–80% confluence.

Cells were divided into three groups:

- Normoxic (C): 95% air/5% CO₂ for 6 h;
- Hypoxia (H): 94% N₂/1% O₂/5% CO₂ for 6 h;
- Hypoxia + inhibitor (Y): pretreated with 5 μM pan‐JNK inhibitor JNK‐IN‐8 (inhibiting JNK1/2/3) for 30 min before hypoxia.

The normoxic and hypoxia groups received DMSO (0.1% v/v) as a vehicle control.

### 2.13 Cellular ATP Content Determination

HT22 cells (1 × 10^6^/well) were exposed to hypoxic (1% O₂) or control conditions for 6 h. The inhibitor group received 5 μM JNK‐IN‐8 for 30 min before hypoxia. After medium removal, samples were lysed with ice‐cold ATP extraction buffer (0.3 mL/well), boiled for 10 min, and centrifuged (10,000 g, 10 min, 4°C). ATP levels were measured by chemiluminescence assay (E–BC‐F002, Elabscience) with a standard curve (*R* ^2^ ≥ 0.999), normalized to protein content, and expressed as μmol/g protein (*n* = 3).

### 2.14 JC‐1 Mitochondrial Membrane Potential Assay

Following the same hypoxic treatment protocol, cells were stained with JC‐1 (10 μg/mL, Beyotime, C2006) at 37°C in the dark for 20 min, washed twice with PBS, and analyzed by flow cytometry (488 nm excitation; FL‐1 and FL‐2 detection). The proportion of polarized mitochondria was calculated as the JC‐1 red‐positive fraction [Q2/(Q2 + Q3)] (mean ± SD, *n* = 3).

### 2.15 Statistical Analysis

Differential metabolites were identified using Wilcoxon rank‐sum test or one‐way ANOVA (VIP > 1.5, fold‐change ≥ 1.5) with FDR‐adjusted *p* < 0.05. Other data were analyzed using GraphPad Prism 10.1. Population data were assessed using Mann–Whitney *U*, chi‐square, Spearman correlation, and Steiger's *Z* tests (online). For animal experiments, normality (Shapiro–Wilk) and homogeneity (Levene's test) were assessed. Two‐group comparisons used *t*‐test or Mann–Whitney *U*; multi‐group comparisons used ANOVA with Tukey HSD (equal variances) or Games‐Howell (unequal variances) post hoc tests, or Kruskal–Wallis with Dunn's test. Statistical significance was set at *p* < 0.05. Data are presented as mean ± SD or median (IQR).

## 3 Results

### 3.1 High‐Altitude Hypoxia Impairs Memory in Native Plateau Residents

Demographic characteristics were comparable between groups (Table S1). Residents at > 4000 m exhibited lower median memory scores than those at 3000–4000 m (4.0 [IQR 2.0–4.0] vs. 5.0 [IQR 3.0–5.0], *p* < 0.0001), with higher prevalence of memory impairment (42.5% vs. 27.9%, OR = 1.91, 95% CI: 1.64–2.24), confirming altitude‐dependent memory vulnerability. Notably, non‐memory scores did not differ (median 22.0, *p* = 0.06), suggesting domain‐specific deficit. Altitude correlated with memory (*r* = −0.165, 95% CI: −0.201 to −0.127, *p* < 0.0001), but not non‐memory performance (*r* = −0.003, *p* = 0.87), with Steiger's *Z* test confirming the significantly stronger altitude‐memory association (*Z* = −6.85, *p* < 0.0001) (Table 1).

**TABLE 1:** Association between altitude and cognitive performance (*n* = 2819).

Outcome	3000–4000 m (*n* = 1339)	> 4000 m (*n* = 1480)	Effect size (95% CI)	*p*
Memory score				
Median (IQR)	5.0 (3.0–5.0)	4.0 (2.0–4.0)	−1.0^a^	< 0.0001
Low memory (≤ 3), No. (%)	373 (27.9)	629 (42.5)	OR = 1.91 (1.64–2.24)	< 0.0001
Non‐memory score				
Median (IQR)	22.0 (20.0–24.0)	22.0 (20.0–23.0)	0.0^b^	0.06
Correlation analysis				
Altitude‐memory correlation	—	—	*r* = −0.165 (−0.201 to −0.127)	< 0.0001
Altitude‐non‐memory correlation	—	—	*r* = −0.003 (−0.041 to 0.035)	0.87
Memory‐non‐memory correlation	—	—	*r* = 0.202 (0.165–0.239)	< 0.0001
Steiger's *Z* (altitude‐memory vs. altitude‐non‐memory)	—	—	*Z* = −6.85	< 0.0001

*Note:* Low memory was defined as a score ≤ 3 points. Correlations were computed using Spearman rank correlation coefficients.

Abbreviations: CI, confidence interval; IQR, interquartile range.

^a^ Median difference from the Mann–Whitney *U* test.

^b^ Median difference from the Mann–Whitney *U* test.

### 3.2 Chronic Hypoxia Impairs Spatial Memory and CA1 Atrophy

**FIGURE 1:**
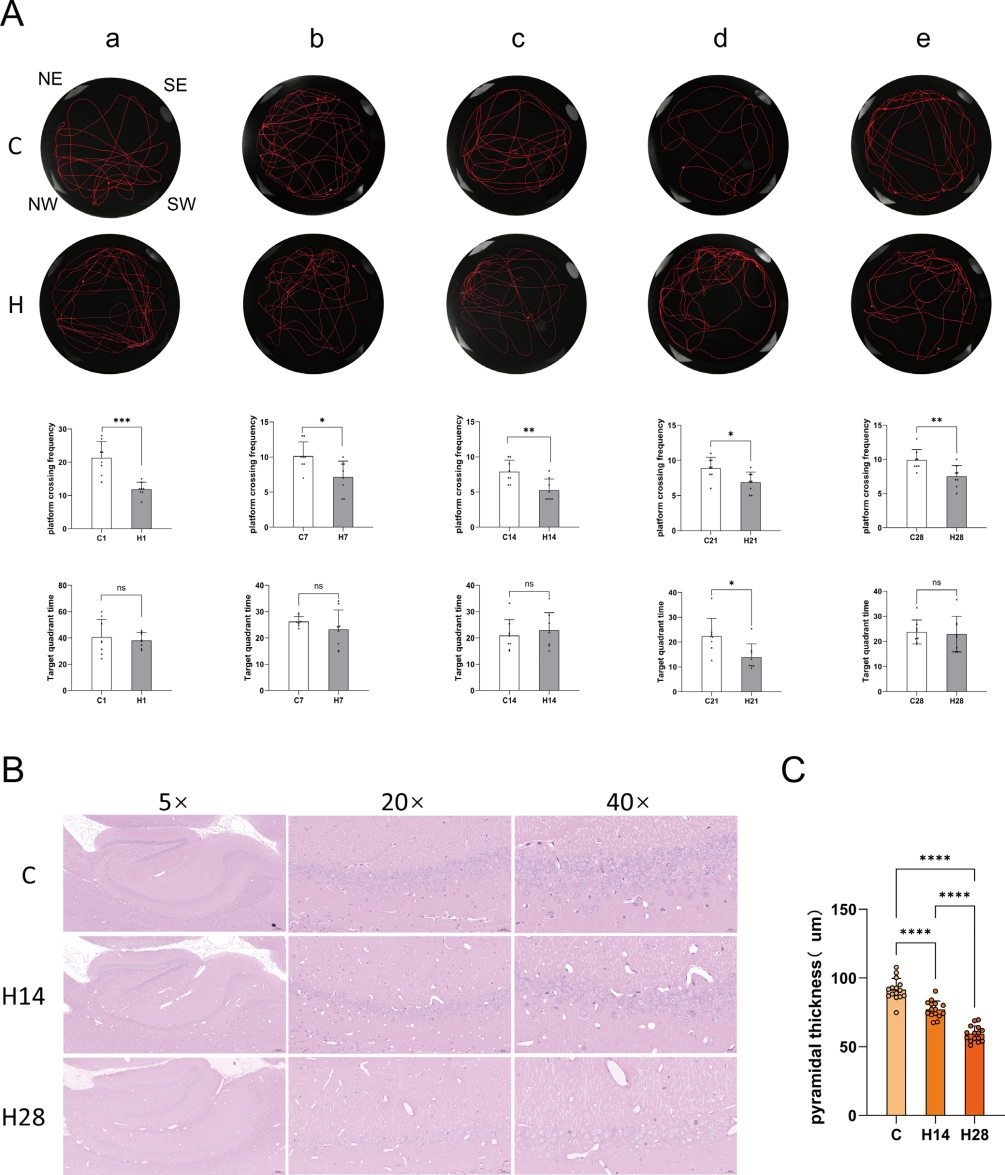
Chronic hypoxia impairs spatial memory and induces hippocampal CA1 atrophy (A) Platform crossing frequency and target quadrant dwell time in the Morris water maze across hypoxia exposure periods (a–e: Days 1, 7, 14, 21, and 28; mean ± SEM; *n* = 8 per group). (B) Representative H&E stained images (5×, 20×, and 40×) showing morphological changes in the CA1 region. Scale bar = 100 μm. (C) Quantification of CA1 pyramidal layer thickness. Data are expressed as mean ± SEM, *n* = 15. C, control group; H, hypoxia group; C1/C7/C14/C21/C28, control groups at indicated days; H1/H7/H14/H21/H28, hypoxia groups at indicated days. *p < 0.05, **p < 0.01, ***p < 0.001, ****p < 0.0001. CA1, Cornu Ammonis 1 (hippocampal subfield); H&E, hematoxylin and eosin; SEM, standard error of the mean.

Chronic hypoxia induced sustained deficits in spatial memory, with platform crossings reduced at all timepoints (Day 1–28; all *p* < 0.05; Figure 1A), whereas target quadrant dwell time differed only at Day 21 (*p* = 0.0182). Concordantly, CA1 pyramidal layer thickness exhibited progressive atrophy from Day 14 through Day 28 (*p* < 0.0001; Figure 1B,C), indicating that structural degeneration underlies sustained memory dysfunction.

### 3.3 Hypoxia Activates JNK‐p53‐Bim Signaling in the Hippocampus

**FIGURE 2:**
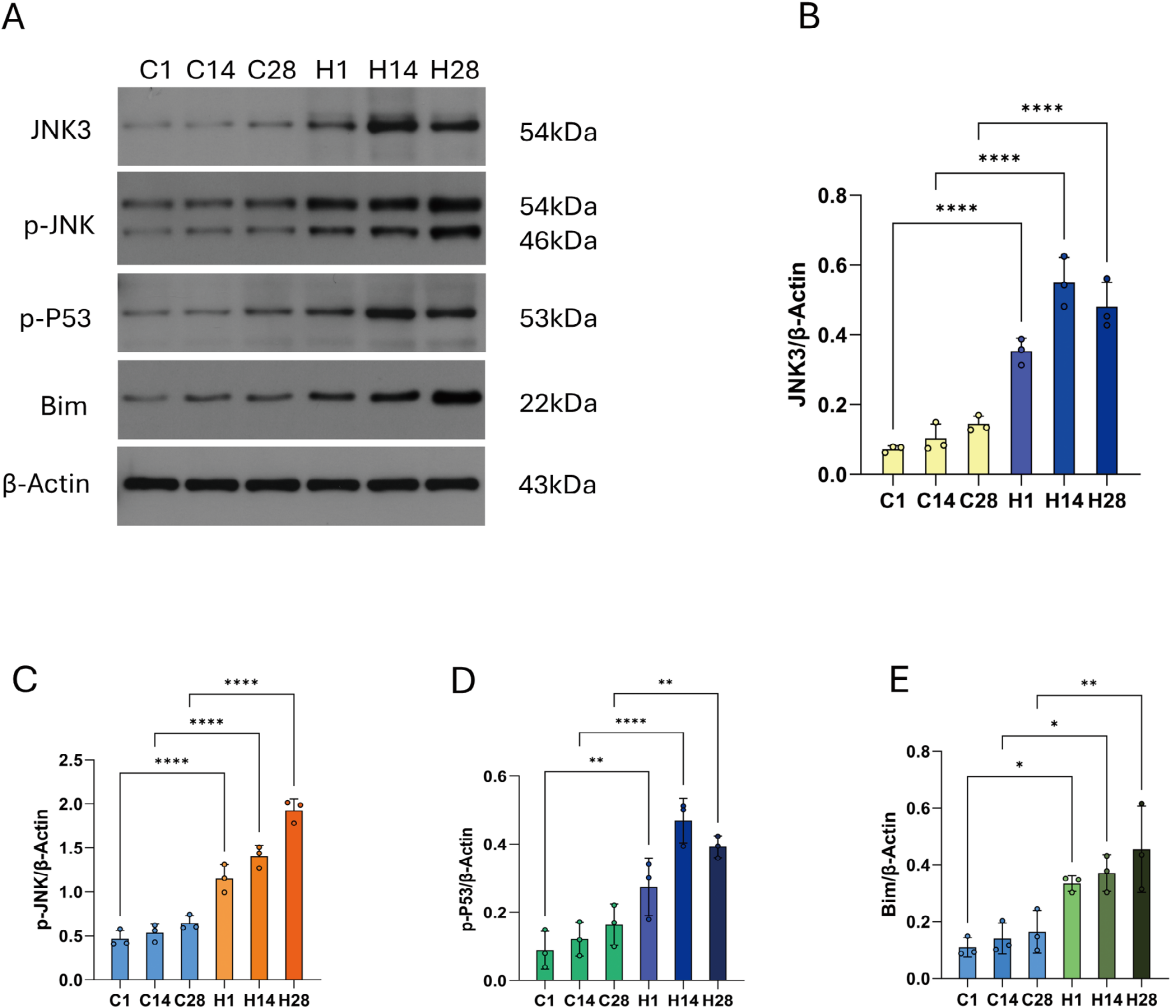
Protein expression levels of JNK3, p‐JNK, p‐P53, and Bim in hippocampal tissue following chronic hypoxia. (A) Representative Western blots of JNK3, p‐JNK, p‐P53, Bim, and β‐Actin. (B–E) Quantification normalized to β‐Actin (mean ± SD, *n* = 3). C1/C14/C28, control groups; H1/H14/H28, hypoxia groups at indicated days. *p < 0.05, **p < 0.01, ****p < 0.0001. Bim, Bcl‐2‐interacting mediator of cell death; JNK3, c‐Jun N‐terminal kinase 3; p‐JNK, phosphorylated JNK; p‐P53, phosphorylated P53; SD, standard deviation.

Western blot analysis revealed significant activation of the JNK‐p53 axis (pan‐JNK phosphorylation, p‐P53, and Bim upregulation), alongside increased total JNK3 protein in hypoxic groups compared with normoxic controls (Figure 2A–E; see Table S2 for detailed densitometry).

### 3.4 Hypoxia Induces Mitophagy‐To‐Apoptosis Transition and OXPHOS Impairment

**FIGURE 3:**
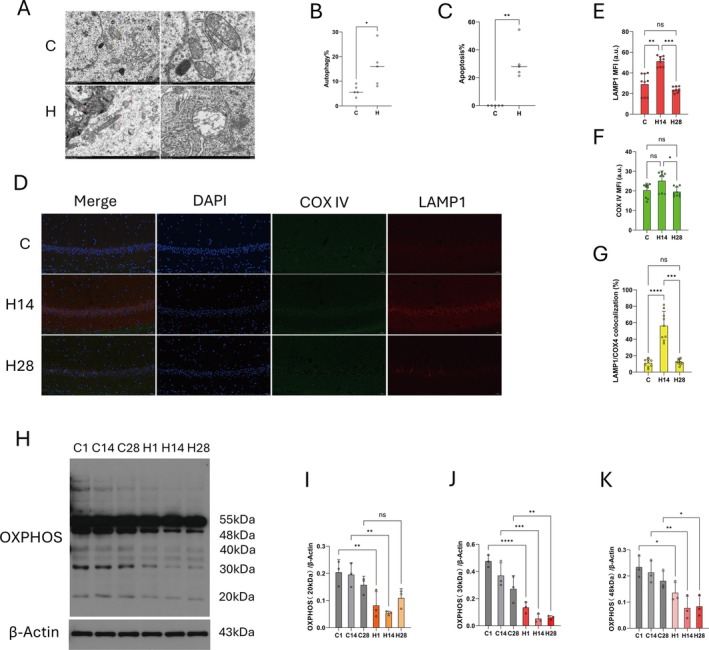
LAMP1–COX4 colocalization and OXPHOS complex expression in the hypoxic hippocampus. (A) TEM images (7000× and 20,000×) of CA1 mitochondria Scale bars = 2 μm and 500 nm. (B) Quantification of mitophagic mitochondria (mean ± SD, *n* = 5). (C) Quantification of apoptotic mitochondria (mean ± SD, *n* = 5). (D) Immunofluorescence of LAMP1 (red) and COX IV (green) in CA1 (20× objective, scale bar = 100 μm). (E–G) Quantification of LAMP1 and COXIV intensities, and colocalization (mean ± SD, *n* = 9). (H) OXPHOS Western blot. (I–K) Complex I (20 kDa), Complex II (30 kDa), and Complex III (48 kDa) quantification (mean ± SD, *n* = 3). C1/C14/C28, controls; H1/H14/H28, hypoxia. *p < 0.05, **p < 0.01, ***p < 0.001, ****p < 0.0001. COX IV, cytochrome c oxidase subunit IV; LAMP1, lysosome‐associated membrane protein 1; OXPHOS, oxidative phosphorylation; OXPHOS, oxidative phosphorylation; SD, standard deviation; TEM, transmission electron microscopy.

TEM revealed pathological progression after 28 days, including autophagic structures, swelling, and cristae disruption (Figure 3A), with quantitative analysis confirming elevated autophagy and apoptosis ratios (*p* < 0.05; Figure 3B,C). Time‐course analysis revealed a biphasic mitophagy response: LAMP1‐COXIV colocalization peaked at Day 14 (56.4% vs. control; *p* < 0.0001) but declined by Day 28 (12.2%; *p* < 0.001), suggesting impaired autophagic flux. Concordantly, OXPHOS complexes exhibited selective vulnerability under hypoxic stress: Complexes I, II, and III were significantly decreased compared to normoxic controls (*p* < 0.05; Figure 3H), whereas Complexes IV and V remained stable, indicating impaired upstream electron transport chain function drives energy.

### 3.5 Metabolic Reprogramming and Energy Crisis Under Chronic Hypoxia

**FIGURE 4:**
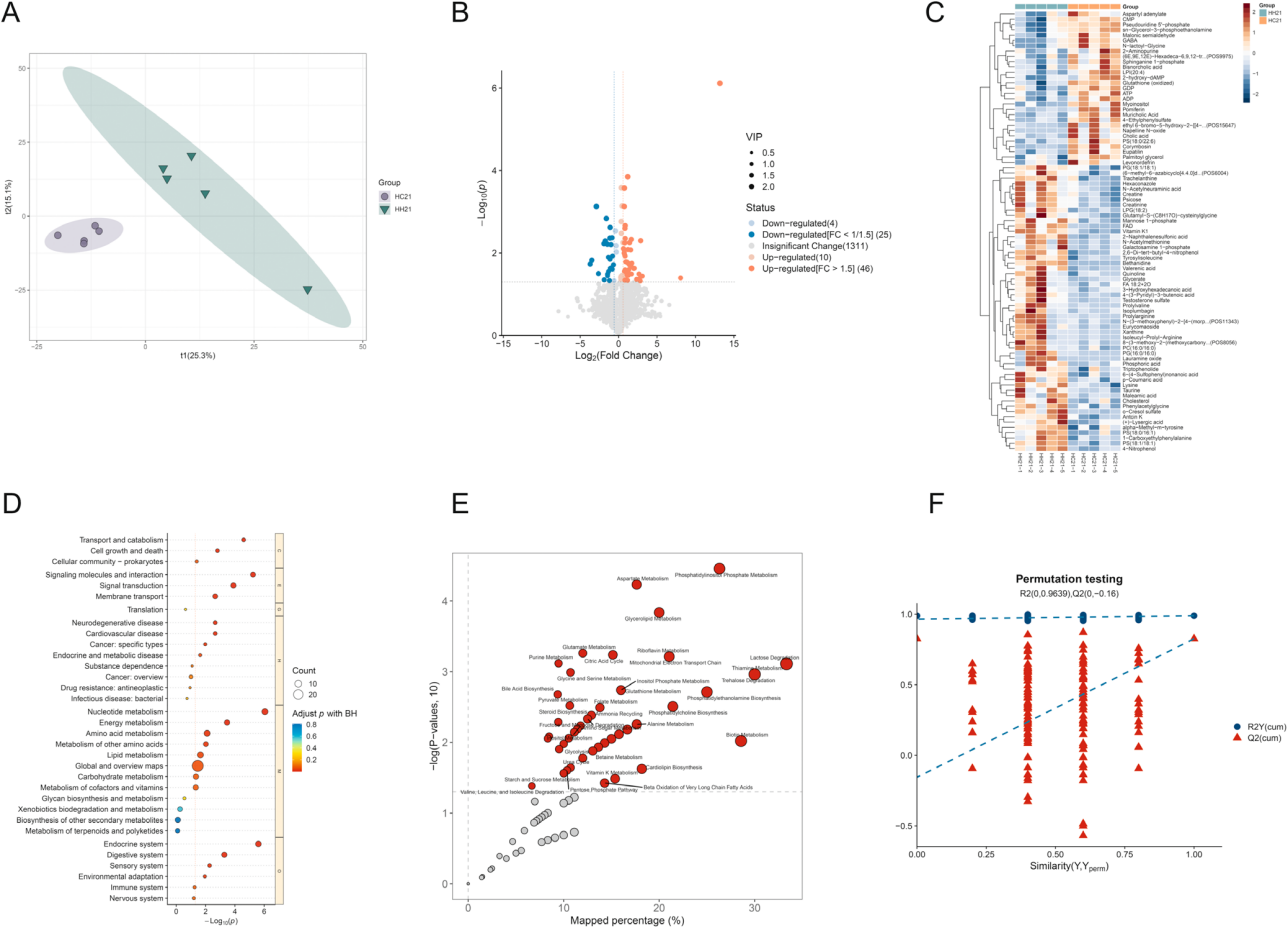
Metabolomic reprogramming under 21‐day hypoxia. (A) PLS‐DA (*n* = 5). (B) Volcano plot. (C) Heatmap. (D) KEGG pathway plot. (E) HMDB pathway scatter plot. (F) Permutation testing validating PLS‐DA model robustness (*n* = 200). *Q* ^2^(cum) = 0.826, *R* ^2^Y(cum) = 0.989, *p* = 0.035. HC21, normoxic control; HH21, 21 day hypoxia. HMDB, Human Metabolome Database; KEGG, Kyoto Encyclopedia of Genes and Genomes; PLS‐DA, partial least squares discriminant analysis; *Q* ^2^, predictive ability parameter; *R* ^2^Y, explained variance in Y.

PLS‐DA revealed robust separation between hypoxia and control groups (*Q* ^2^ = 0.826, *R* ^2^Y = 0.989; permutation test *p* = 0.035; Figure 4A,F), with 83 differential metabolites identified (54 upregulated, 29 downregulated; VIP > 1.5) (Figure 4B). Heatmap analysis showed complete separation between groups (Figure 4C). KEGG and HMDB pathway analysis revealed enrichment of energy, amino acid, and lipid metabolism pathways (Figure 4D,E).

Metabolomic profiling revealed severe disruption of energy homeostasis underlying hypoxic injury. Fourteen energy metabolism‐related metabolites were significantly altered (Table 2). ATP decreased by 73% (*p* = 0.006), concomitant with ADP reduction (FC = 0.47, *p* = 0.0236) and GDP depletion (FC = 0.50, *p* = 0.0063), indicating global high‐energy phosphate pool collapse. Although compensatory mechanisms were activated—including upregulation of glycolytic intermediates (glycerate, malonic semialdehyde, aspartyl adenylate), FAD (1.68‐fold, *p* = 0.0003), phosphocreatine shuttle substrates (creatine and creatinine), and mitochondrial membrane lipids (PC and PG species)—these adaptations proved insufficient to restore ATP homeostasis. Concurrent depletion of nucleotide precursors (2‐hydroxy‐dAMP, 60% decrease) and lactate metabolism intermediates (N‐lactoyl‐glycine, 18% decrease) further underscored the failure of metabolic compensation, confirming irreversible mitochondrial dysfunction drives the energy crisis.

**TABLE 2:** Energy metabolism related differential metabolites in hypoxic versus control brain tissues (*n* = 5).

Metabolite name	FC (H/C)	*p*	PLS‐DA VIP	Pathway/module	Trend
ATP	0.2670	0.0059	1.82	Oxidative phosphorylation	DOWN
ADP	0.4701	0.0236	1.57	Oxidative phosphorylation	DOWN
GDP	0.5026	0.0063	1.76	Purine metabolism/GTP energy pool	DOWN
FAD	1.6839	0.0003	2.28	Electron transport chain	UP
Glycerate	2.5035	0.0255	1.55	Glycolysis	UP
Malonic semialdehyde	0.6285	0.0193	1.58	TCA cycle	DOWN
Aspartyl adenylate	0.3705	0.0327	1.49^a^	TCA anaplerosis	DOWN
N‐lactoyl‐Glycine	0.8138	0.0094	1.71	Glycolysis/Lactate metabolism	DOWN
Creatine	1.7913	0.0119	1.70	Phosphocreatine shuttle	UP
Creatinine	1.7564	0.0113	1.72	Phosphocreatine shuttle	UP
2‐hydroxy‐dAMP	0.4027	0.0282	1.51	Pentose phosphate	DOWN
PC (16:0/16:0)	1.6889	0.0279	1.52	Mitochondrial membrane remodeling	UP
PG (16:0/16:0)	3.1978	0.0324	1.49^a^	Mitochondrial membrane remodeling	UP
PG (18:1/18:1)	1.6561	0.0101	1.68	Mitochondrial membrane remodeling	UP

*Note:* Fold change was calculated as the mean peak area ratio (hypoxia/control). VIP values were obtained from the partial least squares discriminant analysis (PLS‐DA) model. Metabolites were selected based on *p* < 0.05 and VIP > 1.5.

^a^ Two metabolites showed fold changes of 1.49, slightly below the predefined threshold, but were retained because of their biological relevance to energy metabolism.

### 3.6 JNK‐p53 Axis Mediates Hypoxia‐Induced ATP Depletion In Vitro

**FIGURE 5:**
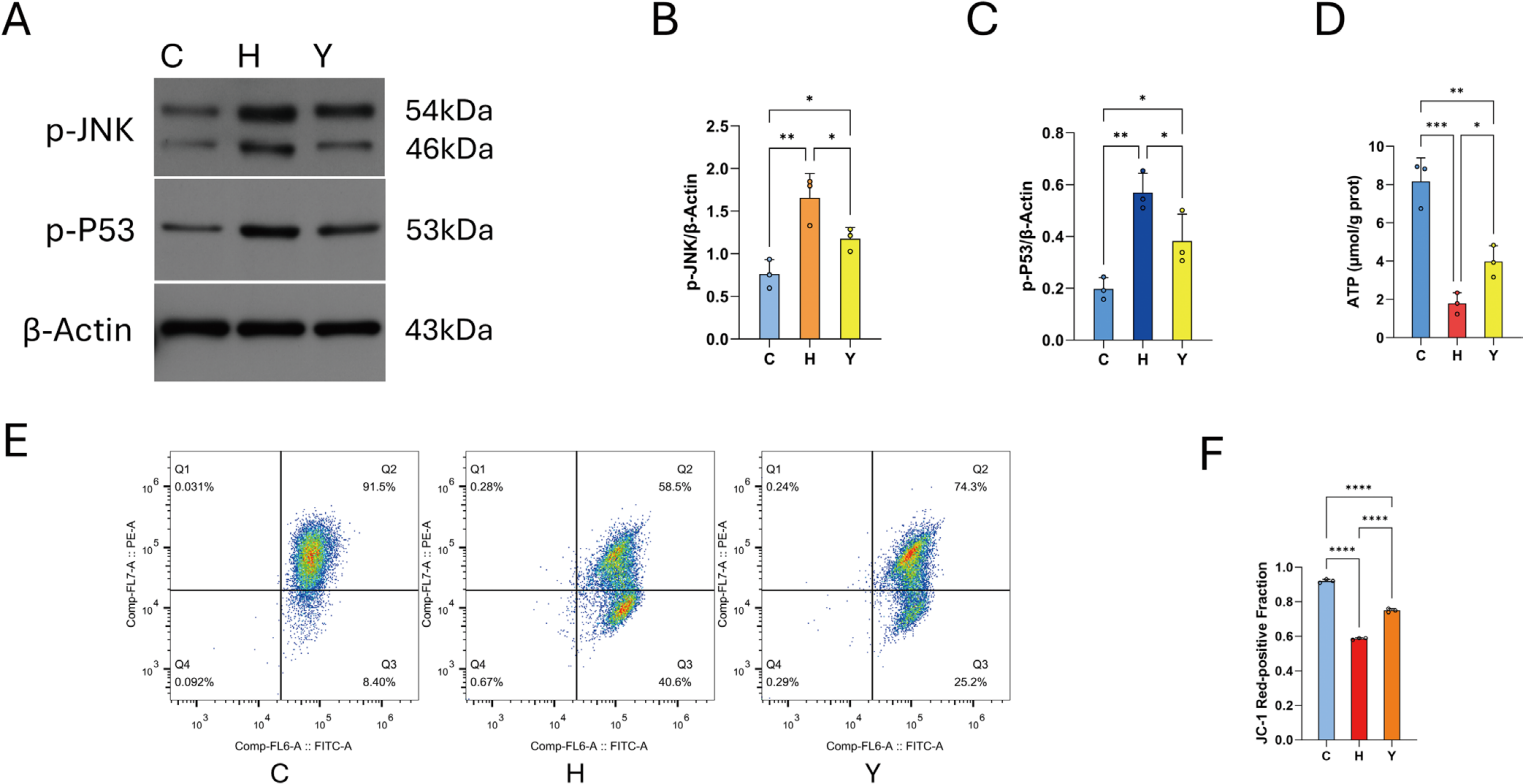
Effects of the JNK inhibitor JNK‐IN 8 on p‐JNK, p‐P53, ATP content, and JC‐1 red‐positive fraction in hypoxic HT22 cells. (A) Western blot analysis of p‐JNK, p‐P53, and β‐Actin. (E) Representative flow cytometry dot plots of JC‐1 staining. Q2, polarized mitochondria (red); Q3, depolarized mitochondria (green). (B–D, F) Quantification (mean ± SD, *n* = 3). C, normoxia; H, hypoxia; Y, hypoxia + JNK‐IN‐8. *p < 0.05, **p < 0.01, ***p < 0.001, ****p < 0.001. JC‐1, 5,5′,6,6′‐tetrachloro‐1,1′,3,3′‐tetraethylbenzimidazolylcarbocyanine iodide; JNK‐IN‐8, specific inhibitor of JNK; p‐JNK, phosphorylated JNK; p‐P53, phosphorylated P53; SD, standard deviation; WB, western blot.

HT22 cells were pretreated with JNK‐IN‐8 or vehicle before hypoxia. Hypoxia markedly activated JNK signaling, evidenced by p‐JNK elevation (0.76 ± 0.17 to 1.66 ± 0.29, *p* = 0.0018), which was attenuated by JNK‐IN‐8 (1.18 ± 0.13, *p* = 0.0288). Concordantly, p‐P53 rose under hypoxia (0.20 ± 0.02 to 0.57 ± 0.07, *p* = 0.0011) and was suppressed by inhibition (0.38 ± 0.10, *p* = 0.0261 vs. hypoxia) (Figure 5A–C). Functionally, ATP depletion (8.17 ± 1.23 to 1.79 ± 0.56 μmol/g protein, *p* = 0.0001) was partially rescued by JNK‐IN‐8 (3.98 ± 0.83, *p* = 0.0267) (Figure 5D), and mitochondrial membrane potential collapse, evidenced by JC‐1 red‐positive fraction reduction (0.92 ± 0.01 to 0.59 ± 0.01, *p* < 0.0001), was partially reversed by JNK‐IN‐8 (0.75 ± 0.01, *p* < 0.0001 vs. hypoxia) (Figure 5E,F), confirming JNK‐p53 inhibition mitigates hypoxic energy crisis.

### 3.7 Adaptive HIF‐2α/PHD2 Signaling Under Chronic Hypoxia

**FIGURE 6:**
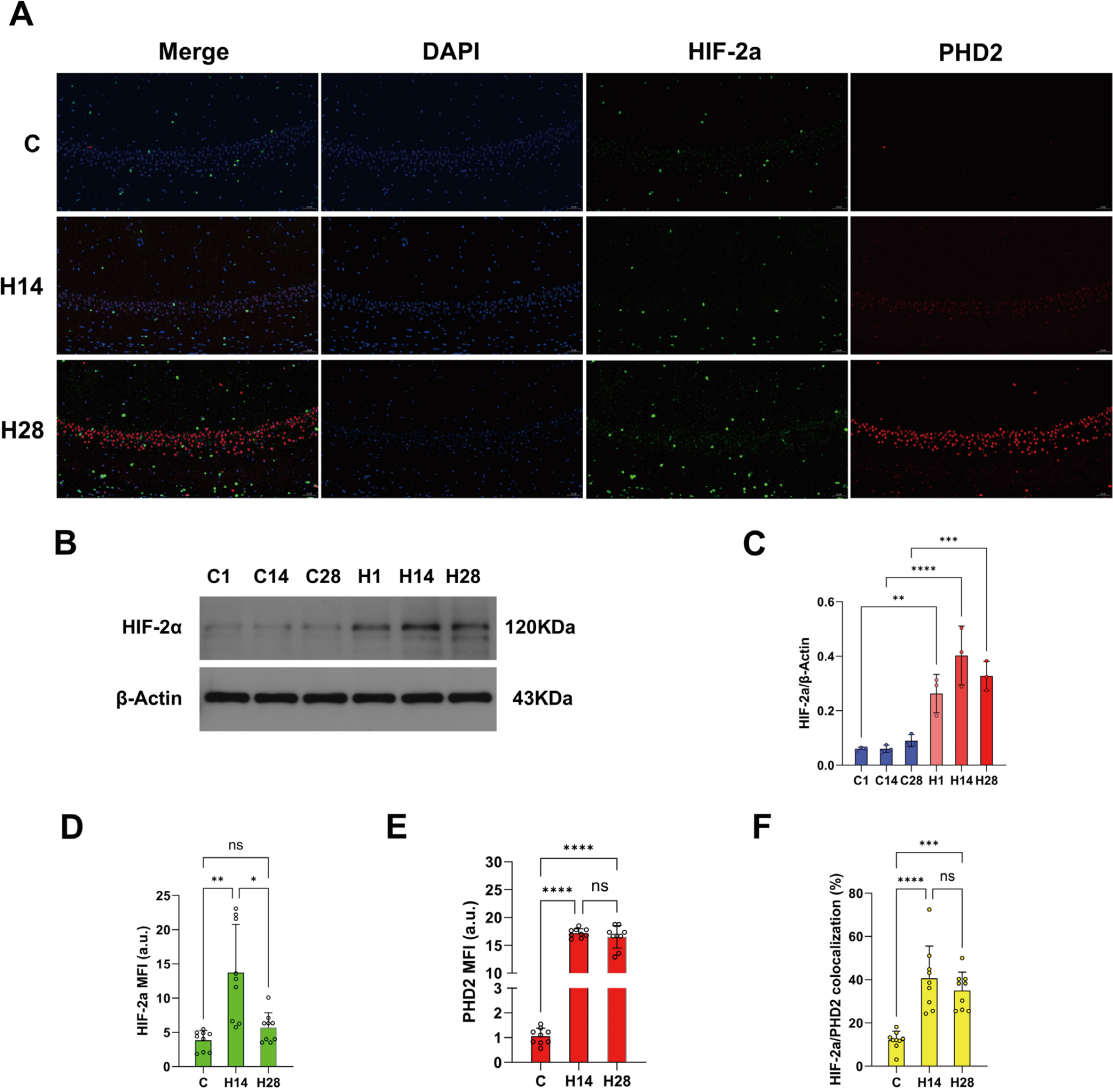
HIF‐2α and PHD2 activation in CA1 under chronic hypoxia. (A) Dual immunofluorescence (scale bar = 100 μm). (B) Western blot of HIF‐2α. (C) Quantification of HIF‐2α protein abundance (mean ± SD, *n* = 3). (D–F) Quantification of PHD2 intensity, HIF‐2α intensity, and colocalization coefficient (mean ± SD, *n* = 9). C1/C14/C28, controls; H1/H14/H28, hypoxia groups. *p < 0.05, **p < 0.01. HIF‐2α, hypoxia‐inducible factor 2 alpha; IF, immunofluorescence; PHD2, prolyl hydroxylase domain protein 2; SD, standard deviation.

Western blot revealed sustained HIF‐2α elevation throughout chronic hypoxia (Day 1, 14, and 28; all *p* < 0.01; Figure 6B,C). Concordantly, immunofluorescence confirmed robust HIF‐2α and PHD2 upregulation in CA1 neurons, with peak HIF‐2α/PHD2 colocalization at Day 14 (40.6%) maintained through Day 28 (35.0%; *p* < 0.001 vs. controls) (Figure 6A,D–F), demonstrating persistent activation of adaptive hypoxic signaling that parallels, yet fails to prevent, the concurrent JNK‐p53‐mediated energy crisis.

## 4 Discussion

This study assessed the population‐level risk of memory impairment across different altitude ranges and investigated the mechanistic role of JNK signaling in driving mitochondrial dysfunction and energy metabolic collapse under chronic hypoxia. We identified a sequential pathogenic cascade wherein pan‐JNK phosphorylation activates p‐P53, upregulates Bim, induces a mitophagy‐to‐apoptosis transition, impairs ATP synthesis, and ultimately results in memory deficits, representing a core mechanism by which hypoxia accelerates neuronal senescence and cognitive decline [17].

Population‐based analyses confirmed that chronic hypoxia impairs memory, with residents living at > 4000 m exhibiting a 91% higher risk than those living at 3000–4000 m. Although hypoxia‐induced memory deficits are reported across species [18, 19], the molecular regulatory axis remained unclear. Using longitudinal animal models, our study systematically identified the JNK‐p53 axis as a key driver.

Chronic hypoxia induced persistent hippocampal damage through Day 28, with synchronized structural degeneration and memory impairment observed by Morris water maze performance and H&E staining. This neuronal degeneration resembles pathological features of senescence, suggesting that chronic hypoxia accelerates brain aging.

The reversibility of high‐altitude hypoxic injury remains controversial. While intermittent or acute hypoxia models often allow functional recovery [20, 21, 22, 23], chronic sustained hypoxia is more likely to induce irreversible damage [24], consistent with the present findings. These discrepancies likely reflect differences in hypoxic intensity, duration, and exposure patterns [25]. Our model reveals the unadulterated injury course, providing insights into chronic hypoxia's impact on energy metabolism and brain aging.

While pan‐JNK phosphorylation was markedly activated, concomitant upregulation of total JNK3 protein suggests neuronal JNK isoforms may contribute to this signaling axis [26, 27, 28, 29]. The pronounced upregulation of JNK3 protein, together with its brain‐restricted expression pattern, implicates JNK3 as a potential, but not exclusive, contributor, though genetic validation is required to establish isoform‐specific roles. In vitro experiments using JNK‐IN‐8 confirmed the causal role of the JNK‐p53 axis, with the inhibitor synchronously suppressing p‐JNK and p‐P53 expression while partially restoring ATP levels to approximately 50% of control values. Safety data from studies of JNK inhibitors in animal models further support translational potential [30, 31]. However, systemic administration of pan‐JNK inhibitors carries potential off‐target toxicity risks, and JNK‐IN‐8 specifically exhibits poor blood–brain barrier penetration. Importantly, favorable safety profiles of JNK inhibitors have been confirmed in early‐phase clinical trials [30, 32], establishing the JNK‐p53 axis as a proof‐of‐concept therapeutic target. Nevertheless, JNK‐IN‐8 currently serves as a pharmacological tool rather than a direct clinical candidate, and future development should prioritize brain‐penetrant, isoform‐selective inhibitors to overcome these pharmacokinetic limitations.

Mitochondria represent the primary subcellular target of the JNK‐p53 axis‐mediated injury. Activation of this pathway induces Bax and Bak activation through Bim upregulation, increasing mitochondrial outer membrane permeability and promoting cytochrome c release to initiate the caspase cascade [33, 34, 35]. Electron microscopy demonstrated that chronic hypoxia induces mitochondrial swelling, cristae disruption, and increased matrix electron density, directly impairing oxidative phosphorylation. Subunits of OXPHOS complexes I, II, and III exhibited a biphasic pattern, characterized by early mild reduction followed by progressive depletion during prolonged hypoxia, whereas complexes IV and V subunits remained relatively stable. JNK‐p53 signaling also contributes to autophagic initiation. JNK‐mediated phosphorylation of Bcl‐2 disrupts the inhibitory Bcl‐2/Beclin‐1 complex, releasing Beclin‐1 to promote autophagosome nucleation [36, 37]. In the specific context of mitochondrial autophagy, p53 transcriptionally upregulates BNIP3 and NIX, while JNK phosphorylation of BNIP3 facilitates LC3 recruitment to damaged mitochondria [38]. These transcriptional and post‐translational inputs suggest that JNK‐p53 functions as an upstream coordinator of the initial mitophagic response, rather than merely a parallel stress signal. Consistently, colocalization of the autophagic marker LAMP1 and COXIV increased at Day 14 but declined by Day 28, indicating early activation of mitophagy followed by impaired autophagic flux and lysosomal dysfunction [39]. This temporal shift coincided with increased apoptosis, supporting a transition from mitophagy‐dominant responses to apoptosis‐dominant injury. In vitro, JNK‐IN‐8 partially reversed both ATP depletion and mitochondrial membrane potential collapse (JC‐1 red‐positive fraction, Figure 5E,F), corroborating that JNK inhibition protects mitochondrial functional integrity. Thus, JNK‐p53‐driven pathology not only promotes neuronal death but may also push surviving neurons toward senescence, establishing this axis as a rate‐limiting determinant of injury progression.

Metabolomic profiling revealed that chronic hypoxia induces compensatory reprogramming of hippocampal metabolic networks but ultimately fails to prevent a severe energy crisis. PLS‐DA demonstrated robust separation of metabolic profiles between hypoxic and control groups, with 14 of 83 differential metabolites associated with energy metabolism pathways [40]. Although upregulation of glycolytic and tricarboxylic acid cycle intermediates indicates activation of both anaerobic and aerobic metabolic processes, ATP levels remained markedly reduced, accompanied by ADP reduction and GDP depletion, reflecting global collapse of the high‐energy phosphate pool due to mitochondrial respiratory chain dysfunction. This dysfunction was supported by the progressive decline of OX‐PHOS complexes I, II, and III, with relative preservation of complexes IV and V, indicating greater vulnerability of upstream electron transport components to hypoxia. Compensatory mechanisms, including activation of the phosphocreatine shuttle, cofactor synthesis, and membrane lipid remodeling, were partially engaged but insufficient to restore ATP homeostasis. This compensation‐to‐decompensation transition represents a hallmark of chronic hypoxia and interacts bidirectionally with JNK‐p53‐mediated mitochondrial damage, forming the metabolic basis of cognitive impairment and further driving cellular senescence [41]. This energetic failure preferentially compromises synaptic function. Synaptic mitochondria are preferentially vulnerable to bioenergetic stress, with compartmentalized ATP decline preceding non‐synaptic failure and correlating directly with spatial memory deficits [42]. At the molecular level, synaptic vesicle glutamate loading depends on ATP‐driven VGLUT activity; ATP crisis disrupts this process, causing excitatory transmission failure and excitotoxicity [43, 44]. Chronic hypobaric hypoxia reduces CA1 synaptic plasticity protein expression, synapse number, and dendritic spine density, paralleling memory decline [39]. The synchronization between ATP decline (Day 21), CA1 atrophy (Days 14–28), and spatial memory deficits (Days 1–28) positions ATP depletion as a proximal energetic mediator. Direct ATP supplementation into hippocampal CA1 rescues Morris water maze performance in energetic stress models [45], establishing that restoring local energy availability is sufficient to ameliorate spatial memory deficits.

Chronic hypoxia concurrently activates HIF‐2α‐mediated adaptive responses and JNK‐p53 axis‐mediated injury pathways. Immunofluorescence analysis revealed that colocalization of HIF‐2α and PHD2 in CA1 neurons peaked on Day 14 and persisted through Day 28, indicating sustained activation of hypoxic adaptive signaling [46], Under normoxic conditions, PHD2 hydroxylates HIF‐2α and promotes its degradation; however, this repression is relieved during hypoxia, enabling HIF‐2α nuclear translocation and subsequent induction of glycolytic reprogramming. Under chronic hypoxic conditions, HIF‐2α signaling interacts with the JNK‐p53 axis, resulting in disruption of mitochondrial architecture and impairment of electron transfer, thereby perpetuating ATP depletion. This imbalance between adaptation and injury is particularly pronounced under persistent high‐altitude hypoxia, where HIF‐2α‐mediated metabolic reprogramming not only fails to alleviate but further exacerbates the energy crisis [41], collectively driving neuronal injury. These findings underscore the biphasic nature of hypoxic responses, in which adaptive and injurious signals coexist, and their antagonistic balance ultimately determines neuronal survival and the progression of senescence.

This study establishes JNK‐p53 inhibition as a potential therapeutic target for chronic high‐altitude hypoxia, reversing mitochondrial decompensation and energy crisis. Pan‐JNK inhibition offers partial neuroprotection and establishes proof‐of‐concept for pharmacological intervention while metabolic biomarkers (ATP/ADP ratio, PC/PG lipids) provide noninvasive assessment tools for future clinical trials.

## 5 Limitations and Future Directions

This study has some limitations. First, pharmacological inhibition with JNK‐IN‐8 targeted pan‐JNK activation without isoform selectivity; therefore, our conclusions pertain broadly to the JNK‐p53 axis. Genetic validation using JNK3‐specific siRNA or knockout models is required to resolve isoform‐specific contributions. Second, metabolomics analysis was performed with *n* = 5 per group as a preliminary screen; larger cohorts are needed to confirm metabolic biomarkers. Third, the HT22 cell line, although hippocampal‐derived, may not fully recapitulate primary neuronal responses. Fourth, while the temporal association between JNK‐p53 activation and the mitophagy‐to‐apoptosis transition supports a regulatory role, our study did not directly test whether JNK inhibition rescues hypoxia‐induced mitophagy (e.g., BNIP3, Parkin, or LC3‐II turnover in inhibitor‐treated animals). Future studies should prioritize development of brain‐penetrant JNK inhibitors, dissection of HIF‐2α and JNK‐p53 crosstalk, and validation of metabolic markers in high‐altitude populations.

## 6 Conclusion

This study demonstrated that chronic high‐altitude hypoxia activates the JNK‐p53 axis, contributing to mitochondrial dysfunction, energy crisis, and cognitive impairment. While HIF‐2α‐mediated glycolytic adaptation provides partial compensation, it is insufficient to prevent progressive hippocampal injury. JNK inhibition represents a potential therapeutic strategy for high‐altitude hypoxic brain injury.

## Funding

This study was supported by the National Natural Science Foundation of China (No. 81974283) and the Science and Technology Project of Qinghai Province (2025‐ZJ‐748).

## Ethics Statement

Human subjects: This study was approved by the Ethics Committee of Qinghai University Medical College (Approval No. P‐SL‐2023‐465). Animals: All animal procedures were approved by the Medical Science Research Ethics Committee of Qinghai University (Approval No. P‐SL‐2023‐465) and conducted in accordance with the Regulations for the Management of Laboratory Animals of Qinghai University (License No. SYXK(Qing)2020–0001).

## Consent

All participants provided written informed consent.

## Conflicts of Interest

The authors declare no conflicts of interest.

## Supporting information


**File S1:** Psychometric Validation of the High‐Altitude Cognitive Function Assessment Scale.

**Table S1:** Baseline characteristics of included participants (*n* = 2819).

**Table S2:** Densitometry data of Western blot analysis for JNK‐p53 axis proteins in rat hippocampus.

## Data Availability

The dataset (Version 3.0) supporting the findings of this study has been deposited in Science Data Bank (https://doi.org/10.57760/sciencedb.33696). All data are available from the corresponding authors upon reasonable request.
