# Supplementary material for: High‐Altitude Hypoxia Activates JNK‐p53 Signaling: Linking Hippocampal Energy Crisis to Cognitive Impairment
Source: CNS Neurosci Ther. 2026 Jun 17;32(6):e70986. doi: 10.1002/cns.70986 (PMC13274233; doi:10.1002/cns.70986)
Supplement: Supplementary file 1 — File S1: Psychometric Validation of the High‐Altitude Cognitive Function Assessment Scale. [file CNS-32-e70986-s003.docx]

**Supplementary File S1**

**Title: Psychometric Validation of the High-Altitude Cognitive Function Assessment Scale**

**Corresponding Authors:** Guoen JIN ([13997030567@163.com](mailto:13997030567@163.com)); Ri-Li Ge ([geriligao@hotmail.com](mailto:geriligao@hotmail.com))

The High-Altitude Cognitive Function Assessment Scale is a culturally adapted cognitive screening instrument independently developed by our research center (National Copyright Registration No. 2024-A-00215453). Due to the limited cultural suitability of the Montreal Cognitive Assessment among native residents of the Tibetan Plateau, which is associated with comprehension barriers and elevated false-positive rates, this scale was specifically designed based on local cultural practices [1].

Psychometric validation in 713 long-term residents demonstrated strong internal consistency, as indicated by a Cronbach's alpha of 0.828, and satisfactory split-half reliability, with a Spearman-Brown coefficient of 0.808 and a Guttman split-half coefficient of 0.754. In a subsample of 111 participants who completed both scales, the high-altitude instrument yielded significantly higher scores than the Montreal Cognitive Assessment (MoCA) [27.0 (23-30) vs. 18.0 (14-24), Z = -8.836, *P* < 0.001], whereas MoCA exhibited a false-positive rate of 61.3% in this population. These results confirm the robust psychometric properties of the scale and its cultural appropriateness for cognitive screening in high-altitude pastoral settings.

**References for Supporting Information**

[1] Hao GS, Ge RL, Jin GE, et al. Modified application of the Montreal Cognitive Assessment Scale in cognitive function screening of native residents on the Qinghai-Tibet Plateau. J High Alt Med Biol. 2025;46(2):118-223.
