# Supplementary material for: High‐Altitude Hypoxia Activates JNK‐p53 Signaling: Linking Hippocampal Energy Crisis to Cognitive Impairment
Source: CNS Neurosci Ther. 2026 Jun 17;32(6):e70986. doi: 10.1002/cns.70986 (PMC13274233; doi:10.1002/cns.70986)
Supplement: Supplementary file 2 — Table S1: Baseline characteristics of included participants (n = 2819). [file CNS-32-e70986-s001.docx]

**Supplementary Table S1**

**Title:** Baseline Characteristics of Study Participants

**For:** High-Altitude Hypoxia Activates JNK-p53 Signaling: Linking Hippocampal Energy Crisis to Cognitive Impairment

**Corresponding Authors:** Guoen JIN ([13997030567@163.com](mailto:13997030567@163.com)); Ri-Li Ge ([geriligao@hotmail.com](mailto:geriligao@hotmail.com))

**Table** S**1** Baseline characteristics of included participants (n=2,819)

| Characteristic | 3,000*-*4,000 m (n=1,339) | >4,000 m (n=1,480) | Statistic | *P* value |
| --- | --- | --- | --- | --- |
| Age, median (IQR), y | 59.0 (53.0*-*68.0) | 60.0 (54.0*-*67.0) | Z = *-*1.75 | 0.08 |
| Sex, No. (%) |  |  | χ² = 0.98 | 0.32 |
| Male | 584 (43.6) | 673 (45.5) |  |  |
| Female | 755 (56.4) | 807 (54.5) |  |  |
| Education level, No. (%) |  |  | χ² = 0.87 | 0.83 |
| Illiterate | 1,175 (87.8) | 1,306 (88.2) |  |  |
| Primary school | 116 (8.7) | 119 (8.0) |  |  |
| Middle school | 30 (2.2) | 38 (2.6) |  |  |
| College and above | 18 (1.3) | 17 (1.2) |  |  |

**Note:** IQR, interquartile range; Continuous variables are presented as median (IQR), and categorical variables are presented as number (percentage). No statistically significant differences in baseline characteristics were observed between groups.
