# Supplementary material for: High‐Altitude Hypoxia Activates JNK‐p53 Signaling: Linking Hippocampal Energy Crisis to Cognitive Impairment
Source: CNS Neurosci Ther. 2026 Jun 17;32(6):e70986. doi: 10.1002/cns.70986 (PMC13274233; doi:10.1002/cns.70986)
Supplement: Supplementary file 3 — Table S2: Densitometry data of Western blot analysis for JNK‐p53 axis proteins in rat hippocampus. [file CNS-32-e70986-s002.docx]

**Supplementary Table S2**

**Title:** Densitometric Analysis of Hippocampal Protein Expression

**For:** High-Altitude Hypoxia Activates JNK-p53 Signaling: Linking Hippocampal Energy Crisis to Cognitive Impairment

**Corresponding Authors:** Guoen JIN ([13997030567@163.com](mailto:13997030567@163.com)); Ri-Li Ge ([geriligao@hotmail.com](mailto:geriligao@hotmail.com))

**Table S2** Densitometry data of Western blot analysis for JNK-p53 axis proteins in rat hippocampus

| Protein | Time Point | Hypoxia (Mean ± SD) | Control (Mean ± SD) | P value |
| --- | --- | --- | --- | --- |
| JNK3 | Day 1 | 0.35 ± 0.04 | 0.07 ± 0.01 | 0.0001 |
|  | Day 14 | 0.55 ± 0.07 | 0.10 ± 0.04 | <0.0001 |
|  | Day 28 | 0.48 ± 0.07 | 0.14 ± 0.02 | <0.0001 |
| p-JNK | Day 1 | 1.15 ± 0.16 | 0.47 ± 0.09 | <0.0001 |
|  | Day 14 | 1.41 ± 0.12 | 0.54 ± 0.10 | <0.0001 |
|  | Day 28 | 1.93 ± 0.13 | 0.64 ± 0.08 | <0.0001 |
| p-P53 | Day 1 | 0.28 ± 0.08 | 0.09 ± 0.06 | 0.0078 |
|  | Day 14 | 0.47 ± 0.07 | 0.12 ± 0.05 | <0.0001 |
|  | Day 28 | 0.39 ± 0.03 | 0.16 ± 0.06 | 0.0016 |
| Bim | Day 1 | 0.33 ± 0.03 | 0.11 ± 0.03 | 0.0136 |
|  | Day 14 | 0.37 ± 0.06 | 0.14 ± 0.06 | 0.0115 |
|  | Day 28 | 0.46 ± 0.15 | 0.16 ± 0.07 | 0.0021 |

**Note:** Values are normalized to β-actin and expressed as mean ± SD (n = 3 per group). Hypoxia exposure was conducted at a simulated altitude of 6,000 m. Control groups were maintained under normoxic conditions at the same time points. Statistical significance was determined by unpaired t-test or Mann-Whitney U test compared with respective controls.
